# Design and Preparation Technology of Green Multiple Solid Waste Cementitious Materials

**DOI:** 10.3390/ma17091998

**Published:** 2024-04-25

**Authors:** Yexin Ge, Xianping Liu, Zhonghe Shui, Xu Gao, Wu Zheng, Zengchao Zhu, Xudong Zhao

**Affiliations:** 1 State Key Laboratory of Silicate Materials for Architectures, Wuhan University of Technology, Wuhan 430070, China; 15725638899@163.com (Y.G.); zheng5@whut.edu.cn (W.Z.); 18700746426@163.com (Z.Z.); xudzhao@polyu.edu.hk (X.Z.); 2 Hubei Heli Jiusheng Concrete Co., Ltd., Wuhan 430200, China; qqcylyy2023@163.com; 3 School of Civil Engineering and Architecture, Wuhan University of Technology, Wuhan 430070, China; x.gao@whut.edu.cn; 4 Department of Civil and Environmental Engineering & Research Centre for Resources Engineering towards Carbon Neutrality, The Hong Kong Polytechnic University, Hong Kong, China

**Keywords:** multiple solid waste cementitious materials, mechanical properties, durability, hydration products, synergistic effect

## Abstract

For solid waste-based cementitious materials, most scholars focus their research on the hydration reaction of cementitious materials, but there is still a lack of solid waste design that comprehensively considers mechanical properties and durability. Therefore, this article focuses on exploring the mix of design and the microscopic and macroscopic properties of multi solid waste cementitious materials (MSWCMs), namely steel slag (SS), slag powder (SP), desulfurization gypsum (DG), fly ash (FA), and ordinary Portland cement (OPC). According to the orthogonal experimental results, the compressive strength of MSWCMs is optimal when the OPC content is 50% and the SS, SP, DG, and FA contents are 10%, 20%, 5%, and 15%, respectively. The MSWCMs group with an OPC content of 50% and SS, SP, DG, and FA contents of 5%, 15%, 5%, and 25% was selected as the control group. The pure OPC group was used as the blank group, and the optimal MSWCMs ratio group had a 28-day compressive strength of 50.7 megapascals, which was 14% and 7.6% higher than the control group and blank group, respectively. The drying shrinkage rate and resistance to chloride ions were also significantly improved, with maximum increases of 22.9%, 22.6%, and 8.9%, 9.8%, respectively. According to XRD, TG-DTG, and NMR testing, the improvement in macroscopic performance can be attributed to the synergistic effect between various solid wastes. This synergistic effect produces more ettringite (AFt) and C-(A)-S-H gel. This study provides a good theoretical basis for improving the comprehensive performance of MSWCMs and is conducive to reducing the use of cement, with significant economic and environmental benefits.

## 1. Introduction

The production of Portland cement accounts for around 7% of total global carbon emissions [1,2], causing significant environmental problems. Meanwhile, inadequate recycling of solid waste is another environmental issue in China, where the production of industrial solid waste is predicted to reach approximately 4.11 billion tons in 2024, with comprehensive utilization amounting to 2.37 billion tons and 890 million tons allocated for disposal. Improper stockpiling or disposal of solid waste not only occupies vast amounts of land but also escalates disposal costs for enterprises, resulting in pollution of surrounding soil, water, and air. Furthermore, it poses hazards such as the potential bursting of dikes and threats to human health [3,4,5]. The utilization of supplementary cementitious materials (SCMs) presents an opportunity to address these challenges by replacing cement and consequently reducing waste and carbon emissions. This approach holds substantial economic and environmental benefits [6,7,8]. Consequently, the resource utilization of solid waste has gained significant attention.

Numerous studies [9,10,11,12] have revealed significant chemical synergistic effects in various solid waste cementitious materials (MSWCMs) systems. There is enormous potential and advantages in using cement as a substitute for cementitious materials, but there are still some issues that need further research, such as the micro- and macroproperties of solid waste cementitious material systems and the relationship between the two [13,14,15]. Long et al. [16] investigated the interface behavior of polymer mortar under different ratios of slag, fly ash, and red mud, using cement mortar as the control group. The consistent results indicated that under different ratios, the compressive strength of slag–fly ash–red mud-based polymer mortar is superior to that of cement mortar. Zhang et al. [17] conducted experimental studies using SP and FA as supplementary cementitious materials. The results showed that the addition of FA and SP delayed the hydration of cement, thereby improving the fluidity of the composite slurry. This also increased the density and integrity of the microstructure of the sample, helping to improve the internal density and compressive strength. Sarıdemir et al. [18] prepared fly ash, slag, silica fume, and metakaolin solid waste cementitious materials, and compared them with ordinary Portland cement. The mechanical properties, microstructure, and durability of the two groups were analyzed. The results showed that fly ash can provide better concrete frames, and its acid and salt resistance are superior to ordinary concrete groups. In addition, some solid waste groups have higher mechanical strength values than ordinary concrete groups. However, in the SEM results, it was found that the problem of concrete cracks caused by the dry shrinkage of solid waste concrete is more serious. Chindaprasir et al. [19] prepared concrete with fly ash–water slurry and silica fume–fly ash solid waste cementitious materials. The results showed that the addition of cement and silica fume can effectively improve the compressive strength of the concrete but significantly reduced its resistance to chloride ions. This is due to the high calcium content of cement. The amount accelerates the decalcification reaction of hydration products, while silica fume breaks the Si-O-Si bond, forming ettringite. Ou et al. [20] prepared steel slag–fly ash and steel slag–silica fume solid waste cementitious materials and investigated the effect of fly ash and silica fume content on the drying shrinkage of concrete. The compressive strength of concrete decreases with the increase in fly ash content, and the addition of silica fume increases the compressive strength of concrete first and then decreases. Both fly ash and silica fume have a positive impact on the drying shrinkage performance of the slurry, but the cost is a different degree of reduction in compressive strength. Park et al. [21] studied the formulation of high-performance concrete with specific bonding materials. The optimal dosage of slag, fly ash, clinker, and dihydrate gypsum was found to be 72%, 10%, 10%, and 8%, respectively. Under long-term sulfate erosion conditions, the strength and strength growth rate of this cementitious material are higher than those of ordinary Portland cement (OPC).

The above literature indicates that many scholars have studied the interactions between various solid wastes. Starting from the binary solid waste cementitious material system, the MSWCMs system has become an increasingly complex system. The reason for this is to continuously compensate for the shortcomings of the previous, more basic system and improve performance. For example, developing a quaternary system from a ternary system may improve the drawbacks of low initial compressive strength and slow setting time. However, currently, most scholars focus their research on the hydration reaction of cementitious materials for multi-component solid waste, and there is still a lack of solid waste design that comprehensively considers mechanical properties and durability. There is relatively little attention paid to the relationship between the synergistic effect of solid waste and the durability of concrete as well as issues such as insufficient strength, poor durability, and susceptibility to cracking in practical applications. Therefore, the complex chemical and physical properties of these materials require in-depth analysis to reveal the relationship between their microstructure and macroscopic properties. In addition, the diversity and uncertainty of solid waste components increase the difficulty of optimizing the mixing ratio, which is key to improving material performance and application scope. At the same time, it can be seen from the above literature that the diversity and complexity of various solid waste raw materials often make it difficult to achieve the mechanical, chloride ion penetration resistance, and drying shrinkage properties of solid waste concrete. This situation limits the performance and widespread application of these innovative materials.

Therefore, in order to overcome these challenges, a reasonable design method was developed to determine the optimal mixing ratio of solid waste cement materials and to improve the mechanical and durability properties of solid waste concrete simultaneously as the central component for improving the utilization rate of solid waste. By precisely adjusting the proportion of each component, the synergistic effect of these materials can be maximized, improving the mechanical properties and durability of concrete while further reducing environmental impact. This method not only helps to optimize the macroscopic properties of materials but also opens up new possibilities for the wider application of these solid waste-based cement materials. Therefore, this article aims to provide theoretical basis and data support for the practical application and production of MSWCMs and to study the feasibility of using SS, SP, DG, and FA as cement substitutes in solid waste cementitious materials. A compressive strength test group for solid waste content was established through orthogonal experiments, and the effects of various solid wastes were studied through multiple factor analysis of variance. The optimal ratio was further determined through optimized ratio design and particle size distribution model validation. Finally, a comprehensive inspection of the micro- and macroperformance of the relevant MSWCMs and OPC systems was conducted, verifying the feasibility of this design preparation method for industrial application reference. It was found to meet the requirements for the comprehensive performance of the MSWCMs system and has huge economic and social benefits.

## 2. Materials and Methods

### 2.1. Materials

The cement used in this study is P-O42.5 OPC, which was sourced from Wuhan Ya Cement Co., Ltd. in Wuhan, China. The solid waste investigated includes SS, SP, DG, and FA. SS and SP were sourced from Wuhan Wuxin New Building Materials Co., Ltd. in Wuhan, China. DG came from Hebei Yousheng Refractory Materials Co., Ltd. in Hebei, China. The FA used is the secondary fly ash provided by Wuhan Qingyuan Power Group Co., Ltd. in Wuhan, China. The FA combustion loss is 4.19%, and the 28-day activity index is 70.3%.

**Figure 1:**
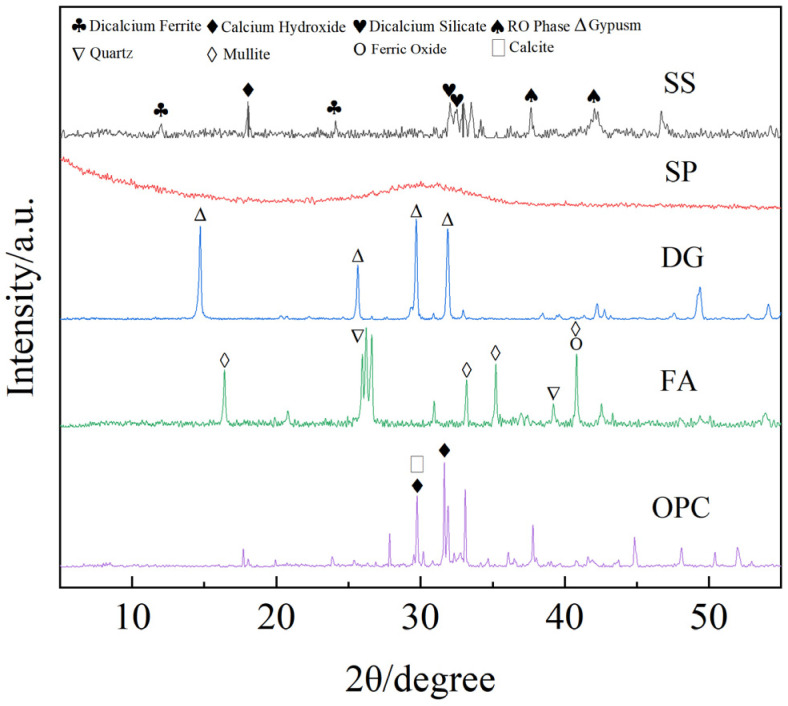
XRD spectra of raw materials.

The volcanic ash effect of solid waste is crucial for it to become a perfect SCM [22,23]. Specific surface area analysis, X-ray diffraction (XRD), X-ray fluorescence spectroscopy (XRF), and thermal analysis are commonly used. XRD is a widely used technical analysis in research. This article quantitatively analyzes the mineral phases present in the raw materials. The XRD spectra of SS, SP, DG, FA, and OPC are presented in Figure 1.

**Figure 2:**
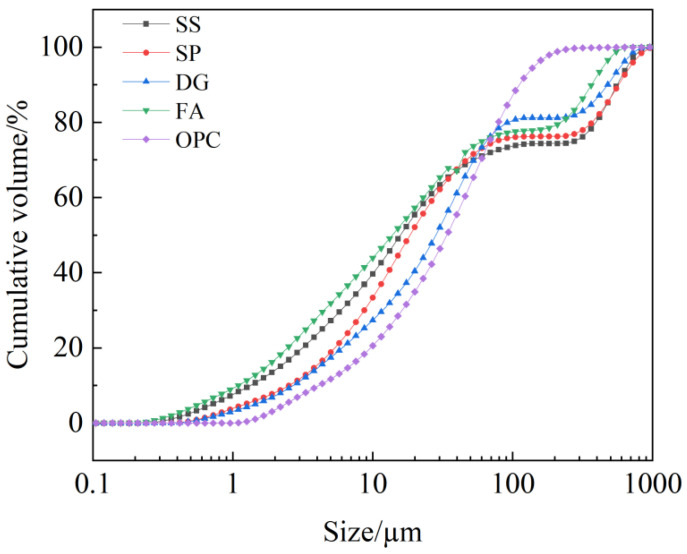
Particle size distribution curve of raw materials.

The analysis results indicate that the primary mineral phases in the FA are mullite and quartz; the main mineral phases in the SS are dicalcium silicate, dicalcium ferrite, calcium hydroxide, and RO phase (solid solutions such as MgO, FeO, and MnO). There is no obvious crystalline phase in the slag, indicating that the SP is mainly in a glassy state. The main mineral phase of desulfurization gypsum is hemihydrate gypsum, and the main mineral phase of the OPC are dicalcium silicate, dicalcium ferrite,.and calcite. XRF can identify the main elements of materials and is a supplementary method to XRD, as it obtains the main elements and elemental elements of the sample and detects the crystal phase, which usually comes from formed compounds. The main chemical components of SS, SP, DG, FA, and OPC are shown in Table 1 and were determined using a PANalytical Axios X-ray fluorescence spectrometer. The particle size of solid waste raw materials is directly related to water demand, workability, and reactivity. The behavior of solid waste raw materials varies at different particle size levels. The particle size distribution of SS, SP, DG, FA, and OPC is shown in Figure 2.

For the fine aggregate, quartz sand, also known as International Organisation for Standardisation (ISO679) standard sand, was utilized [24]. This sand is produced in Xiamen, Fujian, China, and is commonly used as a fine aggregate in various applications.

### 2.2. Mixture Proportions

Based on research findings from domestic and international sources and in accordance with the Chinese standard GB/T 12957-2005 [25], a total of 25 sets of three-factor five-level orthogonal tests were designed using the orthogonal testing principle. In these experimental mixture proportions, five levels of mass proportioning for SS and SP were utilized, namely 0%, 5%, 10%, 15%, and 20%. Additionally, five levels of mass proportioning for DG were used, namely 0%, 2.5%, 5%, 7.5%, and 10%. The remainder was replaced by FA. Quaternary solid waste cementitious materials were used to replace 50% of the mass fraction of OPC to prepare a multiple solid waste cementitious material (MSWCM). The mixture proportions test fit is shown in Table 2. The water/cement ratio used in the test system was 0.4, and the binder/sand ratio was 1:2.

### 2.3. Test Methods

#### 2.3.1. Flowability

The flowability of the composite mortar was determined using the standard “Cement-Based Self-Leveling Mortar for Floor” (JC/T 985-2017) [26]. The maximum diameter of the mixture in two or three different directions was recorded as the fluidity.

#### 2.3.2. Compressive Strength

To examine the mechanical properties, compression strength tests were conducted on the composite mortar specimens using the DYE-300A flexural and compression integrated machine. The equipment is from Beijing North Jianyi Technology Co., Ltd. in Beijing, China.

#### 2.3.3. XRD

After pulverizing and screening the paste specimens, the powdered samples were dried for 8 h in a vacuum-drying oven at 60 °C. Using an X-ray diffractometer (Empyrean) manufactured with a Cu Kα X-ray source under the conditions of 40 kV and 40 mA, XRD was used to characterize the mineral compositions of composite paste specimens. The equipment is from Brucker AG in Karlsruhe, Germany.

#### 2.3.4. TG-DTG

Thermogravimetric testing of the composite paste specimens was performed using a Discovery TGA thermogravimetric analyzer. Among them, the heating rate was 10 °C/min, and the temperature range was 20–1000 °C. The equipment is from NETZSCH Scientific Instruments Ltd. in Selb, Germany.

#### 2.3.5. NMR

The silicon spectrum testing of the composite paste specimens was carried out using a Bruker 600 MHz nuclear magnetic resonance spectrometer. The equipment is from Brucker AG in Karlsruhe, Germany.

#### 2.3.6. Drying Shrinkage

The standard “Dry Shrinkage Test Method of Cement Mortar” (JC/T 603-2004) was used for the dry shrinkage test [27]. The drying shrinkage value was the arithmetic average of the test values of the three specimens.

#### 2.3.7. Chloride Permeation Resistance

The RCM-NTB chloride diffusion coefficient tester was used for the rapid chloride migration (RCM) test. The equipment is from Beijing Shourui Datong Technology Co., Ltd. in Beijing, China.

## 3. Results and Discussion

### 3.1. Flowability

**Figure 3:**
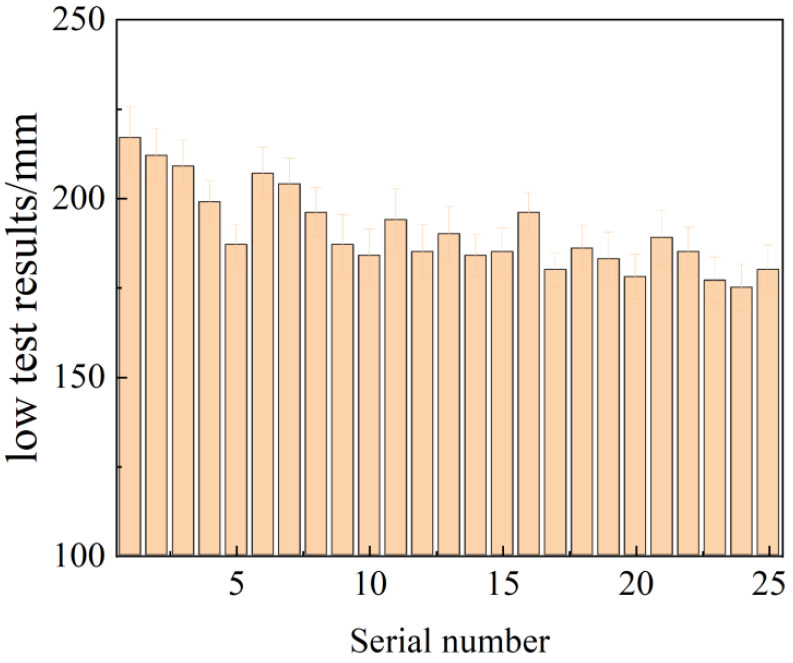
Flow test results of composite mortar.

Figure 3 displays the results of the flowability test conducted on the composite mortar.

From the figure, it can be observed that the dosage of FA exhibits a positive correlation with the fluidity of the composite mortar, with a more pronounced effect. This can be attributed to the finer particle size distribution of the FA, which contributes to the “micro-aggregate effect” of the FA. This effect enhances the particle gradation of the collodion material, facilitating the filling of particles and allowing water to flow between them, thereby significantly improving the fluidity of the slurry. Furthermore, the relatively smooth surface of FA, characterized by spherical particles, helps reduce friction and further enhances the fluidity of the mortar. On the other hand, the effect of other solid wastes on flowability was found to be insignificant. Thus, it can be concluded that increasing the dosage of FA effectively improves the flowability of the slurry [28], while the influence of other solid wastes on flowability is minimal.

### 3.2. Mechanical Properties

#### 3.2.1. Compressive Strength

**Figure 4:**
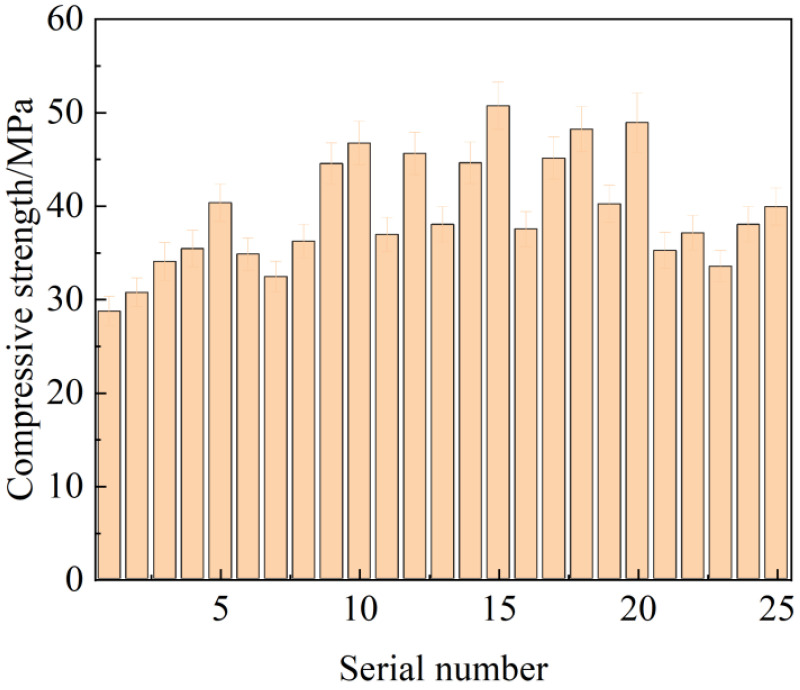
Compressive strength of composite mortar at 28 d.

Figure 4 presents the compressive strength results of the composite mortars after 28 days. It can be seen that the composite mortar has the highest compressive strength of 50.7 MPa when the MSWCMs composition content is SS:SP:DG:FA:OPC = 10:20:5:15:50.

As mentioned earlier, when the optimal design ratio of multiple solid wastes is achieved, their synergistic reaction is maximized, resulting in superior mechanical properties. Comparatively, the compressive strength of the OPC group under the same conditions was 47.1 MPa, while the optimal ratio group achieved a strength of 50.7 MPa, surpassing that of the OPC group. This indicates a noticeable synergistic effect among the solid wastes, leading to a significant advantage in terms of mechanical properties. The improvement in mechanical behavior can be attributed to the following factors:

Physical effect: The filling effect of FA particles plays a crucial role here. Reacted and unreacted FA particles fill the micropores, especially when finer FA particles are added, leading to a denser-filling stacking structure. This significantly enhances the fluidity and mechanical properties of the slurry.

Chemical effect: The synergistic effect of multiple solid wastes influences the hydration kinetics of the system. The exceptional performance of cementitious materials composed of multiple solid wastes can be attributed to the higher generation of ettringite and C-(A)-S-H gel. The formation of C-(A)-S-H gel is crucial for the densification of the mortar pore structure, thereby positively influencing strength development. Although FA has a negative effect on mechanical properties, the alkaline environment provided by SS promotes the volcanic ash reaction of FA, mitigating this negative effect.

It is important to note that the level of FA replacement should not exceed 20% in this study. High levels of FA replacement can have a dilution effect on the paste and lead to delayed hydration of Al. Consequently, the compressive strength of FA slurries with high replacement levels typically tend to be poor.

#### 3.2.2. Orthogonal Test Analysis

To enhance the mechanical properties, an orthogonal test was employed to optimize the proportion of cementitious materials. The optimization process involved using the dosages of SS, SP, DG, and FA as input variables, with each raw material having a specified dosage range as constraints. The aim was to maximize the strength ratio of the cementitious materials.

As can be seen from Table 3, a multi-factor analysis of variance was used to study the influence of SS, SP, and DG on the mechanical properties of mortar instead of FA [29].

From the above table, it can be seen that SS presents significance (F = 12.375), and SP also presents significance (F = 10.5080); this indicates that the main effects of both SS and SP have a substantial impact on the relationship and yield noticeable differences in the results. Conversely, the significance of DG (F = 3.115) is comparatively lower, suggesting that its effect on the relationship and results is relatively less pronounced.

**Figure 5:**
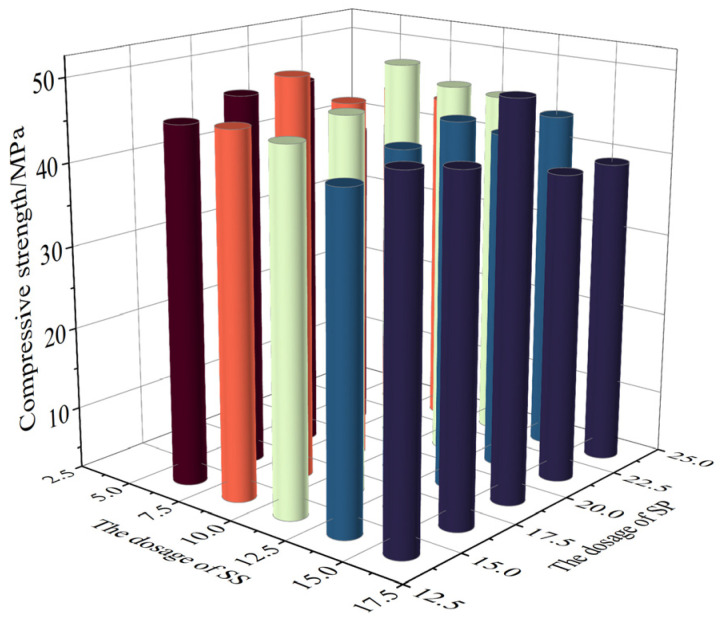
Compressive strength of proportionally optimized composite mortar at 28 d.

According to the results of ANOVA, the design of the SS mass admixture is 5%, 7.5%, 10%, 12.5%, and 15%; of the SS mass admixture is 15%, 17.5%, 20%, 22.5%, and 25%; of the DG mass admixture is 5%; and of the FA mass admixture is 15%. By replacing 50% of the mass fraction of OPC with the MSWCMs, an MSWCM was prepared. The compressive strength results are presented in Figure 5.

From Figure 5, it can be observed that the optimum compressive strength for the MSWCMs is achieved when SS doping is 10%, SP doping is 20%, DG doping is 5%, FA doping is 15%, and OPC doping is 50%. We selected the MSWCMs group with the OPC content of 50% and SS, SP, DG, and FA contents of 10%, 20%, 5%, and 15%, respectively, as the optimal ratio group and named it as the SS10SP20 group based on the SS and SP content. Next, we selected the MSWCMs group with the OPC content of 50% and SS, SP, DG, and FA contents of 5%, 15%, 5%, and 25%, respectively, as the control group, and named it the SS5SP15 group. The pure OPC group was selected as the blank group, and the optimal MSWCMs ratio group had a 28-day compressive strength of 50.7 megapascals, which was 14% and 7.6% higher than the control group and blank group. Furthermore, microstructural analysis was conducted on the SS10SP20, SS5SP15, and OPC groups, along with durability tests, to establish a correlation between the chemical composition of the materials and their performance. 

#### 3.2.3. Granularity of Cementitious Materials

As is well known, the addition of solid waste admixtures has a significant impact on the performance of cementitious materials. The changes are primarily attributed to the particle size distribution effect and particle filling effect of solid waste raw materials. The microstructure characteristics of particles in the MSWCMs system, such as different appearances, total surface area, and cumulative distribution of particle size, are quite different from those in the OPC system, thus having a significant impact on the slurry performance. Thus, by adjusting the particle size distribution of cementitious materials and increasing the mass percentage of small particle sizes, the best uniform and dense stacking can be achieved. This can minimize the gaps between particles as much as possible, thereby achieving the goal of reducing the required water consumption and improving the fluidity and other properties of the slurry.

At present, in the current research on the gradation of cementitious materials, the particle size distribution of cementitious materials that conforms to the ideal sieve analysis curve (referred to as Fuller curve) is the mathematical expression shown in Equation (1):

(1) $$A=100\times {\left(\frac{d_{i}}{D}\right)}^{0.4}$$

A—Passing amount through standard sieve analysis, %;
d_i_—Size of each classification of standard sieve holes, mm;
D—The particle size of the largest particle in the combined aggregate, mm;
0.4—Index of the shape characteristics of aggregate particles.

**Figure 6:**
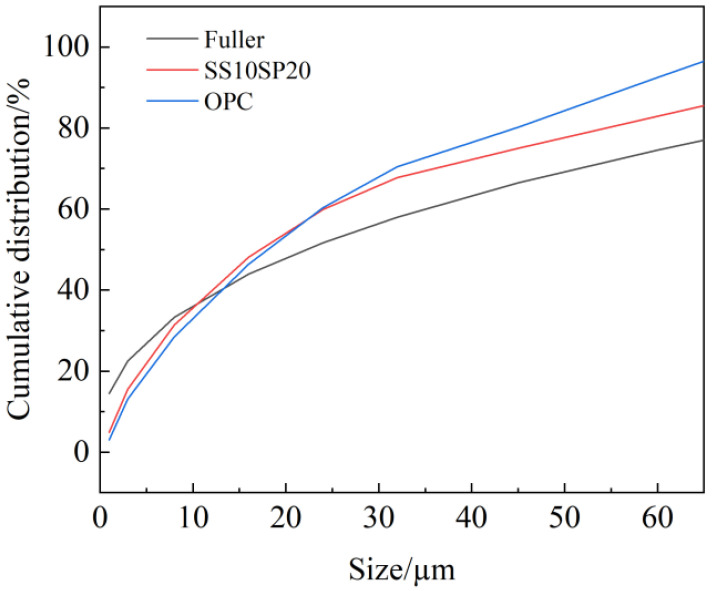
Particle size distribution curve of cementitious materials.

Figure 6 displays the particle size distribution curves of the SS10SP20 group and the OPC group. By adjusting and calculating the gradation curve of the cementitious material system composed of MSWCMs, it approximates the Fuller curve distribution. The figure displays the comparison between the particle distribution curves of the two groups and the Fuller curve. Compared with the OPC group curve, the SS10SP20 group curve is more in line with the Fuller curve distribution, with the smallest area difference between the two, thus achieving the best uniform and dense stacking to a certain extent, which yields an important improvement in the performance of MSWCMs.

### 3.3. Durability

#### 3.3.1. Drying Shrinkage

**Figure 7:**
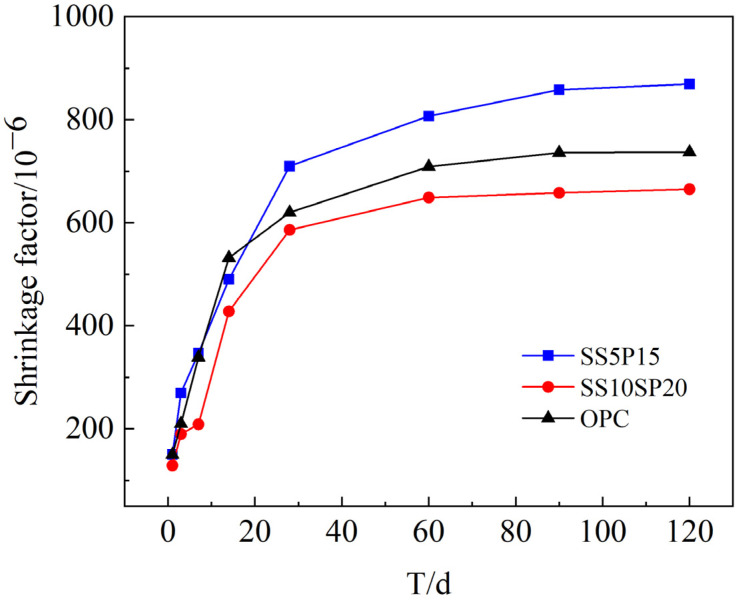
Drying shrinkage coefficient of composite mortar.

Drying shrinkage refers to the rapid loss of free water in cementitious materials, leading to self-drying and a reduction in macroscopic volume. The hydration reaction of mortar consumes a significant amount of water and generates a large number of pores within the system. Some of these pores form curved liquid surfaces, creating internal stresses. As the material ages, significant shrinkage deformations occur [30,31]. Figure 7 illustrates the test results of the drying shrinkage properties for the SS10SP20 group, SS5SP15 group, and OPC group.

From Figure 7, it can be observed that the drying shrinkage coefficient follows the order of the SS5SP15 group > OPC group > SS10SP20 group. This indicates that the drying shrinkage performance of the SS10SP20 group is superior to that of the OPC group, representing a 8.9% improvement in drying shrinkage performance. The lower hydrophilicity of the particles in the SCMs present in the cementitious material compared to the OPC group contributes to the deterioration of water retention and increased water secretion in the mortar. This leads to the formation of capillary pore channels within the specimen, facilitating water evaporation. However, the addition of DG promotes the production of AFt during the hydration process of the mortar [32], and this production is accelerated. The poorer drying shrinkage performance of the SS5SP15 group can be attributed to the incomplete synergistic reaction among the multiple solid components. This incomplete reaction results in increased water absorption in the mortar, leading to a significant loss of mortar quality.

#### 3.3.2. Chloride Permeation Resistance

**Figure 8:**
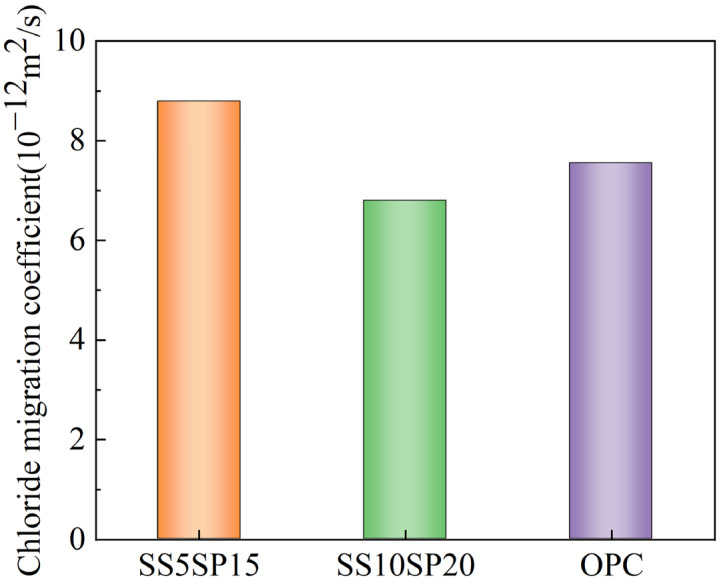
Chloride ion migration coefficient of composite mortar.

Chloride ion penetration is a significant factor contributing to concrete corrosion, and the RCM method is widely employed for assessing the resistance of cementitious materials to chloride ion attack [33]. Figure 8 displays the results of the chloride ion penetration resistance tests conducted on the SS10SP20, SS5SP15, and OPC groups, and as can be seen from the figure, the SS10SP20 group exhibits a 9.8% increase in chloride ion resistance compared to the OPC group.

The presence of Ca(OH)_2_ crystals in mortar hydration products is the primary cause of concrete erosion. These crystals are coarse, unstable, and prone to chemical reactions with harmful ions. The MSWCMs exhibits excellent initial conservation ability. Through the secondary hydration of solid waste and the main hydration product Ca(OH)_2_ in the system, the volcanic ash effect reduces the production of Ca(OH)_2_ crystals. Additionally, the formation of C-(A)-S-H gel and other secondary hydration products rapidly fills the internal pore network. These secondary hydration products significantly enhance the mortar’s ability to conserve chloride ions. Consequently, the penetration rate of chloride ions into the mortar is reduced, leading to improved resistance against chloride ion penetration. Furthermore, fine-grained FA particles contribute to improving the pore structure in the cementitious material to a certain extent. The reactivity of volcanic ash in the MSWCMs plays a crucial role in enhancing the mechanical properties and resistance to chloride ion penetration in cement mortar, which is consistent with previous studies [34,35].

Therefore, the incorporation of MSWCMs can modify the hydration rate and products of the cementitious materials, ultimately improving its resistance to chloride ion penetration. This explains the superior resistance to chloride ion penetration observed in the SS10SP20 group compared to the OPC group. Additionally, the lower reactivity of FA reduces the impact of volcanic ash on the formation of hydration products and the improvement in pore structure. However, the effect of volcanic ash still contributes to a reduction in the production of Ca(OH)_2_ crystals. Consequently, the mortar of the SS5SP15 group, which contains a higher FA content, exhibits lower resistance to chloride ion penetration. Within a certain range of substitution levels, the utilization of SCMs such as SS and SP to replace cement can increase their resistance to chloride ion penetration.

### 3.4. Microstructural Analysis

#### 3.4.1. XRD Analysis

**Figure 9:**
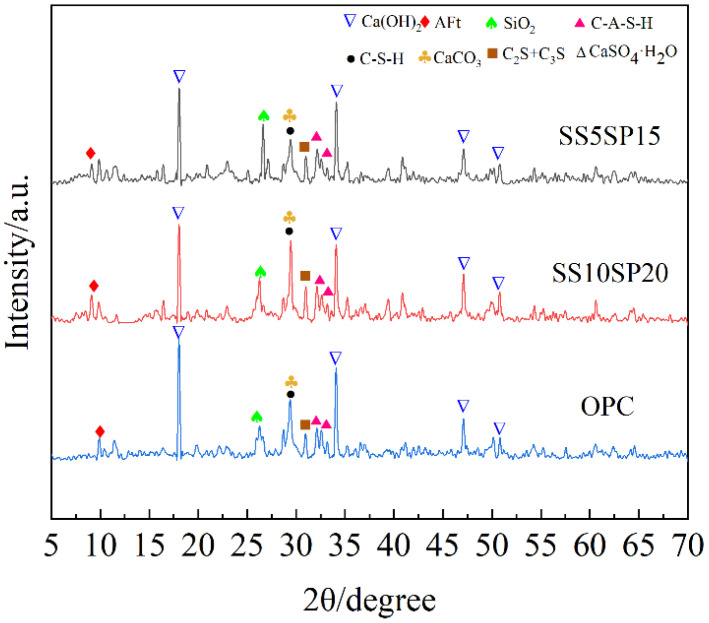
XRD spectra of composite paste at 28 d.

The XRD results of the paste samples from the SS10SP20, SS5SP15, and OPC groups after 28 days are presented in Figure 9.

Based on the figure, it can be observed that the main hydration products in each group include calcium hydroxide (Ca(OH)_2_), AFt, C-(A)-S-H gel, calcite (CaCO_3_), as well as dicalcium silicate (C_2_S) and tricalcium silicate (C_3_S), which did not participate in the hydration reaction. The diffraction peak intensities of AFt were found to be highest in the SS10SP20 group, followed by the SS5SP15 group, and finally the OPC group. This indicates that the SS10SP20 group generated a greater amount of AFt. The presence of DG in the system, on one hand, provides a suitable amount of SO_4_^2−^, and on the other hand, FA serves as the nucleation site for AFt, promoting its formation. In contrast, the SS5SP15 group exhibits a diffraction peak of gypsum, suggesting that the added FA did not fully react and did not contribute to a favorable synergy within the system, resulting in the production of fewer gelling materials.

Additionally, from the diffraction peak intensity shown in Figure 8, it can be observed that the main hydration product of each group of specimens is calcium hydroxide Ca(OH)_2_, which promotes hydration and the continuous release of hydroxyl groups, thereby creating an alkaline environment for the entire system. The Ca(OH)_2_ in the OPC group is significantly higher compared to the SS10SP20 and SS5SP15 groups. DG provides the system with a significant amount of Ca^2+^ and SO_4_^2−^, while FA primarily releases Al^3+^. This leads to the formation of a liquid phase rich in ions such as [Al(OH)]_6_^3−^, Ca^2+^, SO_4_^2−^, [H_3_SiO_4_]^−^, and OH^−^. These ions further react in the liquid phase to produce C-(A)-S-H gel and AFt.

Furthermore, the intensity of SiO_2_ diffraction peaks in the SS5SP15 group is noticeably higher than in the SS10SP20 group. This indicates that the synergistic reaction among the raw materials in the SS5SP15 group is incomplete, resulting in lower production of C-(A)-S-H gel and AFt. This finding aligns with the previous observation of the compressive strength pattern.

It is worth mentioning that diffraction peaks of CaCO_3_ were detected in all XRD results. This occurrence can be attributed to the partial carbonation of Ca(OH)_2_ during the curing process, leading to the formation of a calcite phase.

#### 3.4.2. TG-DTG Analysis

**Figure 10:**
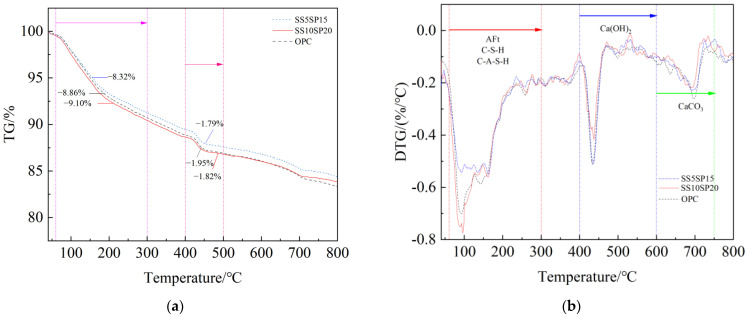
TG-DTG curves of composite paste at 28 d. (**a**) TG; (**b**) DTG.

The TG-DTG curves of the paste samples from the SS10SP20, SS5SP15, and OPC groups at the age of 28 days are displayed in Figure 10.

In each group, three distinct decomposition peaks are observed in the DTG curves within the temperature range of 60–750 °C. The first decomposition peak, occurring within the range of 60–300 °C, corresponds to the mass loss of bound water in the hydration products of C-(A)-S-H gel and AFt. The mass loss rates for this peak were measured to be 9.10%, 8.32%, and 8.86% for the SS10SP20, SS5SP15, and OPC groups, respectively. The second decomposition peak, observed at 400–500 °C, is attributed to the thermal decomposition of Ca(OH)_2_, with mass loss rates of 1.82%, 1.79%, and 1.95% for the SS10SP20, SS5SP15, and OPC groups, respectively. The third decomposition peak, existing within the range of 600–750 °C, is associated with the thermal decomposition of CaCO_3_ resulting from carbonization [36]. The mass loss rates for this peak were measured to be 1.81%, 1.91%, and 2.01% for the SS10SP20, SS5SP15, and OPC groups, respectively.

Quantitative analysis of the thermogravimetric curve revealed that the mass loss rate of C-(A)-S-H gel and AFt follows the order of SS10SP20 group > OPC group > SS5SP15 group. This indicates that the MSWCM in the SS10SP20 group exhibits a favorable synergistic effect. In comparison to the OPC group and SS5SP15 group, the SS10SP20 group produces a greater amount of C-(A)-S-H gel and AFt. This is beneficial for the development of the mechanical and durability properties of the mortar, which is in line with the findings from the XRD analysis.

#### 3.4.3. NMR Analysis

**Figure 11:**
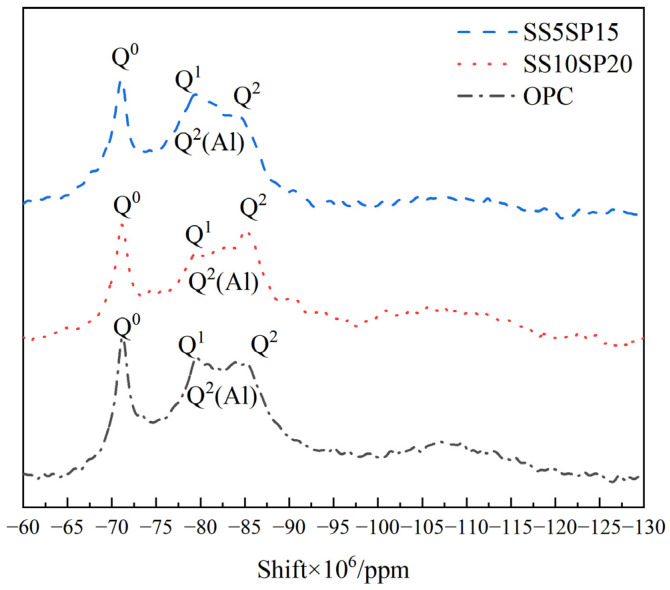
^29^ Si MAS NMR spectra of composite paste at 28 d.

The ^29^Si NMR spectra of the composite paste from the SS10SP20, SS5SP15, and OPC groups are presented in Figure 11.

^29^Si NMR analysis provides insights into the relative number of silicon atoms in different Q^n^ (n is 0 to 4) tetrahedral environments, where n represents the number of bridging oxygens between each silica–oxygen tetrahedron [37]. Q^n^ represents, respectively, Q^0^ (silica–oxygen tetrahedrons), Q^1^ (silica–oxygen tetrahedrons at the end of the straight chain in the C-S-H dimer or in the polymer), Q^2^ (silica–oxygen tetrahedrons in the middle of the straight chain of C-S-H), and Q^2^ (Al) (silica–oxygen tetrahedrons in the middle of the C-S-H chain adjacent to 1 aluminum–oxygen tetrahedron).

As can be seen in Figure 10, in the paste specimens of the SS10SP20, SS5SP15, and OPC groups, and the signal intensities of Q^2^ as well as Q^2^ (Al) are SS10SP20 group > OPC group > SS5SP15 group. This indicates that the synergistic reaction is more complete in the SS10SP20 group, generating more gels. One of the peaks near −82 × 10^−6^~−84 × 10^−6^ ppm corresponds to Q^2^ (Al), which indicates the presence of silica–oxygen tetrahedrons in the middle of the C-S-H chain adjacent to one aluminum–oxygen tetrahedron. This peak is mainly observed due to the substitution of aluminum by silicon, shifting the signal from 3 × 10^−6^~5 × 10^−6^ ppm to a more positive value. The Q^2^ (Al) peaks in all three groups are observed as small shoulders, indicating the formation of C-A-S-H during the hydration process of the system. The intensity of Q^2^ (Al) in the SS10SP20 group is the highest, suggesting that more C-A-S-H is generated in this group.

## 4. Conclusions

In this study, the following main conclusions were drawn through experimental research and theoretical analysis:

1. The optimal mixing ratio of MSWCMs was selected based on the results of an orthogonal test, and the content of optimal ratio components was SS:SP:DG:FA:OPC = 10:20:5:15:50 by using ANOVA, and its reasonableness was verified by the optimized ratios and particle gradation. The results of the optimized ratios were in accordance with the results of the ANOVA, and the distribution curves of the particles also exhibited a high degree of consistency with the Fuller curve, which indicated that the results of the orthogonal test group had a high degree of precision;
2. The composition content of the control group was SS:SP:DG:FA:OPC = 5:15:5:25:50, and the pure OPC group was the blank group. The 28-day compressive strength of the MSWCMs optimal proportioning group was 50.7 MPa, while that of the control group and the blank group was 44.5 MPa and 47.1 MPa, respectively, which showed an increase of 14% and 7.6% in comparison with that of the control and the blank group, indicating a significant increase in the 28-day compressive strength of MSWCMs of 7.6%, which represents a significant advantage;
3. The designed mortar with the optimal proportion of MSWCMs showed excellent durability properties compared to the control and blank mortars. The synergistic effect between the MSWCMs increased the drying shrinkage and resistance to chloride ion permeability of the mortar by 22.9%, 22.6% and 8.9%, 9.8%, respectively. These findings demonstrate the ability of MSWCMs to effectively improve the good durability of large admixture solid waste concrete as well as their great potential as a novel construction material;
4. The mortar in the optimal proportioning group of MSWCMs showed significant synergistic effects compared to the mortar in the control and blank groups. It produced more C-(A)-S-H gels and AFt. The C-(A)-S-H) gels had a higher degree of cross-linking, leading to a denser microstructure. This contributes to improved mechanical and durability properties;
5. The optimal MSWCMs prepared by orthogonal experimental design were also shown to be stronger than other MSWCMs as well as OPC systems in terms of microscopic properties and macroscopic properties after the optimized ratio design and particle grading model verification, which demonstrated the feasibility of this design and preparation method to satisfy the requirements for the comprehensive performance of the MSWCMs system, which has great economic and social benefits.

## Figures and Tables

**Table 1:** Main chemical composition of raw materials.

Materials	Mass Fraction/%									
	CaO	SiO_2_	Al_2_O_3_	Fe_2_O_3_	SO_3_	MgO	Na_2_O	K_2_O	MnO	Others
OPC	53.83	23.92	6.93	3.29	2.39	1.76	0.16	0.91	0.03	6.78
SS	45.97	12.36	1.90	26.11	0.36	4.31	0.12	0.00	3.07	5.8
SP	39.80	32.05	16.71	0.37	2.63	6.60	0.35	0.45	0.17	0.87
DG	36.76	2.27	0.81	0.48	47.44	1.14	0.13	0.00	0.01	10.96
FA	4.58	46.65	36.31	3.93	1.26	0.64	0.39	1.00	0.05	5.19

**Table 2:** Test mixture proportions.

Serial Number	Mass Fraction/%					Water/Cement Ratio	Binder/Sand Ratio
	SS	SP	DG	FA	OPC		
1	0	0	0	50	50	0.4	1:2
2	0	5	2.5	42.5	50	0.4	1:2
3	0	10	5	35	50	0.4	1:2
4	0	15	7.5	27.5	50	0.4	1:2
5	0	20	10	20	50	0.4	1:2
6	5	0	10	35	50	0.4	1:2
7	5	5	0	40	50	0.4	1:2
8	5	10	2.5	32.5	50	0.4	1:2
9	5	15	5	25	50	0.4	1:2
10	5	20	7.5	17.5	50	0.4	1:2
11	10	0	7.5	32.5	50	0.4	1:2
12	10	5	10	25	50	0.4	1:2
13	10	10	0	30	50	0.4	1:2
14	10	15	2.5	22.5	50	0.4	1:2
15	10	20	5	15	50	0.4	1:2
16	15	0	5	30	50	0.4	1:2
17	15	5	7.5	22.5	50	0.4	1:2
18	15	10	10	15	50	0.4	1:2
19	15	15	0	20	50	0.4	1:2
20	15	20	2.5	12.5	50	0.4	1:2
21	20	0	2.5	27.5	50	0.4	1:2
22	20	5	5	20	50	0.4	1:2
23	20	10	7.5	12.5	50	0.4	1:2
24	20	15	10	5	50	0.4	1:2
25	20	20	0	10	50	0.4	1:2

**Table 3:** ANOVA table.

Source of Variation	Square Sum	Degrees of Freedom	Mean Square	F-Value
Intercept	38,659.424	1	38,659.424	5205.441
SS	367.638	4	91.909	12.375
SP	312.170	4	78.042	10.508
DG	92.538	4	23.134	3.115

## Data Availability

The data presented in this study are available on request from the corresponding authors. The data are not publicly available due to ongoing study.
